# Supplementary material for: Autonomic Dysfunction Contributes to Impairment of Cerebral Autoregulation in Patients with Epilepsy
Source: J Pers Med. 2021 Apr 17;11(4):313. doi: 10.3390/jpm11040313 (PMC8073240; doi:10.3390/jpm11040313)
Supplement: Supplementary file 1 [file jpm-11-00313-s001.zip › jpm-1173226-supplementary.pdf]

Journal of Personalized Medicine

MDPI

Table S1. Modified composite autonomic scoring scale

	Modified Composite Autonomic Scoring Scale (mCASS)
**Sudomotor**	(Q-sweat volume)
**0**	Normal
**1**	Any of the following:
	Single Q-Sweat site abnormal
	Length-dependent pattern (distal sweat volume < 1/3 of proximal value)
	Persistent sweat activity at foot
**2**	Any of the following:
	Single site < 50% of lower limit
	Two or more sites reduced
**3**	Two or more sites < 50% of lower limit
**Cardiovagal**	(Deep breathing + Valsalva heart rate)
**0**	Normal
**1**	HRDB mildly reduced but > 50% of minimum
**2**	HRDB reduced to < 50% of minimum or HRDB + VR reduced
**3**	Both HRDB and VR reduced to < 50% of minimum
**Adrenergic**	(Valsalva + Tilt BP)
**0**	Normal
**1**	Any of the following:
	Valsalva maneuver:
	Phase IIe reduction < 40 or > 20 mmHg MBP (30-40 if > 50 years)
	Phase II $_L$ does not return to baseline
	Pulse pressure reduction to ≤ 50% of baseline
	HUT:
	Excessive oscillations in MBP (> 20mmHg)
	Fall in pulse pressure > 50%
	Transient fall in SBP > 20mmHg with recovery within 1 min
	Systolic BP reduction ≥ 20 mmHg beyond 1 min
	Diastolic BP reduction ≥ 10 mmHg beyond 1 min
	Overshoot ≥ 20 mmHg following tilt back
**2**	Any of the following:
	Valsalva maneuver:
	Phase IIe > 40 mmHg MBP
	Score of 1 from Valsalva maneuver + HUT:
	Transient fall in SBP >30 mmHg, recovery within 2 min
	Systolic BP reduction ≥20 mmHg beyond 1 min
	Diastolic BP reduction ≥ 10 mmHg beyond 1 min
**3**	Valsalva maneuver:
	Phase IIe reduction > 40 mmHg + absent II $_L$ and IV
**+1**	HUT:

SBP reduction ≥ 30 mmHg beyond 2 min, sustained for ≥ 2 min

Abbreviations: HRDB, heart rate response to deep breathing; VR, Valsalva ratio; BP, blood pressure; MBP, mean blood pressure; HUT, head up tilt; SBP, systolic blood pressure; DBP, diastolic blood pressure.

Table S2: The comparison of the serial mean arterial pressure, middle cerebral artery velocity and heart rate detected in 5 min head-up tilting tests in patients with epilepsy and the controls.

Time of Recorded		MAP (mmHg)	MCAv (cm/sec)	HR (bpm)	CVR (mmHg × sec/cm)
Baseline	Controls	86.0 (77.3, 97.5)	62.0 (49.0, 83.0)	70.0 (64.5, 74.5)	1.32 (1.08, 1.93)
	Patients	89.3 (82.3, 96.8)	64.0 (50.0, 82.0)	72.0 (64.5, 76.5)	1.39 (1.04, 1.86)
	p value	0.186	0.712	0.449	0.705
1st min	Control	93.0 (83.5, 102.2)	57.0 (48.5, 71.0)	76.0 (70.0, 82.0)	1.58 (1.21, 2.02)
	Patients	91.3 (85.5, 98.7)	62.0 (44.0, 76.0)	79.0 (69.0, 86.0)	1.60 (1.19, 2.05)
	p value	0.665	0.916	0.406	0.792
2nd min	Controls	93.0 (83.3, 101.0)	59.0 (47.0, 74.0)	77.0 (71.5, 86.5)	1.54 (1.26, 2.06)
	Patients	93.7 (84.8, 99.2)	60.0 (43.5, 79.0)	80.5 (72.0, 87.0)	1.35 (1.20, 1.46)
	p value	0.968	0.721	0.561	0.004*
3rd min	Controls	92.0(83.7, 99.0)	59.0 (47.0, 75.0)	80.0 (75.0, 90.0)	1.51 (1.21, 2.02)
	Patients	93.0 (83.3, 100.8)	60.5 (45.2, 81.2)	81.0 (73.2, 90.0)	1.53 (1.12, 2.03)
	p value	0.785	0.693	0.928	0.932
5th min	Controls	94.3 (84.0, 102.0)	59.0 (46.0, 73.0)	82.0 (77.0, 89.0)	1.50 (1.28, 2.02)
	Patients	92.7 (83.8, 98.7)	58.0 (41.0, 80.0)	80.5 (72.0, 91.0)	1.65 (1.17, 2.16)
	p value	0.493	0.956	0.510	0.897
Ending	Controls	87.0 (80.8, 95.7)	61.0 (48.0, 78.5)	67.0 (61.5, 74.5)	1.37 (1.06, 1.98)
	Patients	88.3 (82.7, 94.8)	64.0 (45.5, 78.5)	70.0 (64.5, 76.0)	1.40 (1.05, 2.02)
	p value	0.560	0.692	0.121	0.593

Values are presented as median (IQR).

Abbreviations: MAP, mean arterial pressure; MCAv, middle cerebral artery velocity; HR, heart rate; CVR, cerebrovascular resistance.

*$p < 0.05$ means significant difference between the patients with epilepsy and the controls using the Mann-Whitney U test.
